# Stevioside protects against acute kidney injury by inhibiting gasdermin D pathway

**DOI:** 10.1002/SMMD.20240010

**Published:** 2024-06-28

**Authors:** Ruochen Qiao, Hui Wang, Dasheng Li, Yu Yang, Jiaxin Shu, Xiang Song, Xiaozhi Zhao, Li Lu

**Affiliations:** ^1^ Institute of Translational Medicine the Affiliated Drum Tower Hospital of Nanjing University Medical School Nanjing Jiangsu China; ^2^ University College London School of Pharmacy London UK; ^3^ Hospital‐Acquired Infection Control Department Nanjing Drum Tower Hospital The Affiliated Hospital of Nanjing University Medical School Nanjing Jiangsu China; ^4^ Department of Andrology Nanjing Drum Tower Hospital The Affiliated Hospital of Nanjing University Medical School Nanjing Jiangsu China; ^5^ Department of Andrology Nanjing Drum Tower Hospital The Affiliated Hospital of Nanjing University of Chinese Medicine Nanjing Jiangsu China

**Keywords:** acute kidney injury, gasdermin D, inflammation, stevioside

## Abstract

Recent studies indicate a significant upregulation of gasdermin D (GSDMD) in acute kidney injury (AKI), a severe medical condition characterized by high morbidity and mortality globally. In this study, we identified and validated the therapeutic effects of small molecule inhibitors targeting the GSDMD pathway for AKI treatment. Using a drug screening assay, we evaluated thousands of small molecules from DrugBank against Lipopolysaccharide (LPS) and Nigericin‐stimulated immortalized bone marrow‐derived macrophages (iBMDMs) to discern GSDMD pathway activators. We simulated AKI in primary renal tubular epithelial cells using hydrogen peroxide (H_2_O_2_) exposure. Furthermore, AKI in mouse models was induced via cisplatin and ischemia/reperfusion. Our findings highlight stevioside as a potent GSDMD activator exhibiting minimal toxicity. Experimental results, both in vitro and in vivo, demonstrate stevioside's significant potential in alleviating renal tubular epithelial cell injury and AKI histological damage. After stevioside treatment, a notable decrease in cleaved GSDMD‐N terminal levels was observed coupled with diminished inflammatory factor release. This observation was consistent in both cisplatin‐ and ischemia/reperfusion‐induced AKI mouse models. Collectively, our research suggests that stevioside could be a promising candidate for modulating GSDMD signaling in AKI treatment.

## Graphical abstract

Stevioside, a natural compound identified in our screenings, was shown to markedly prevent cisplatin‐induced cell death in HK‐2 cells, exhibiting a clear concentration‐dependent protective effect. Post‐injury treatment with stevioside led to a decrease in GSDMD expression and cleaved GSDMD‐NT levels, curtailing the release of inflammatory cytokines and reducing apoptosis and reactive oxygen species. In AKI mouse models, induced by both cisplatin and ischemia‐reperfusion, stevioside treatment demonstrated significant renal protection, mitigating kidney damage markers, marking the first study to elucidate stevioside's therapeutic potential in AKI through GSDMD pathway inhibition.

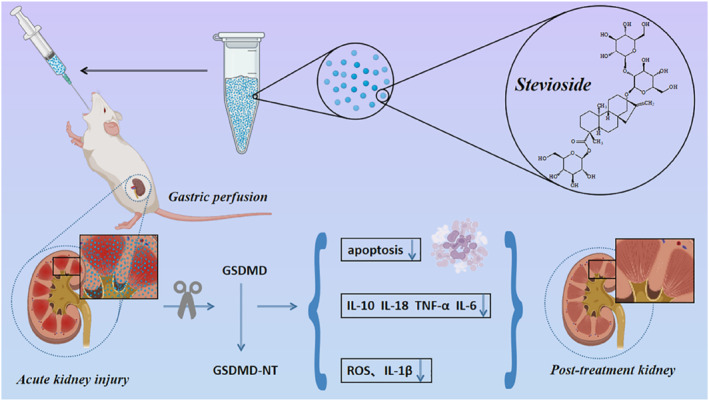

**Key points**

- Our research has found that stevioside, a natural compound, can significantly reduce the release of inflammatory factors.
- By inhibiting the activation of the GSDMD signaling pathway, stevioside prevents cell pyroptosis and enhances renal protection.

## 1 INTRODUCTION

Acute kidney injury (AKI) presents as a widespread issue manifesting both in community and hospital settings.^1^ AKI arises due to a myriad of causes, including ischemia, toxic injuries, and other pathogenic influences, and is linked with both short‐term and long‐term morbidity and mortality.^2^, ^3^, ^4^, ^5^ Clinically, AKI patients display an abrupt decline in renal function, marked by a pronounced increase in serum creatinine (SCr) levels. This clinical picture is further characterized by azotemia, disturbances in water‐electrolyte and acid‐base balances, and systemic symptoms.^6^, ^7^ Contemporary clinical solutions are limited to supportive measures such as renal transplantation and renal dialysis, but neither effectively reverses AKI.^6^, ^8^ Hence, there's a pressing need for efficacious therapeutic drugs and strategies.

Inflammatory factor release is emblematic of AKI.^9^ The renal tubules, pivotal in AKI pathology, also serve as significant sources of these inflammatory cytokines.^10^ Current literature has identified notable upregulation of caspase‐11 and gasdermin D (GSDMD) in AKI models induced by both cisplatin and ischemia‐reperfusion (IR).^11^, ^12^ Here, caspase‐11 acts to cleave GSDMD, producing the GSDMD‐N terminal (GSDMD‐NT). This terminal then forms membrane pores, facilitating cell lysis, leading to the release of a vast number of inflammatory factors and thereby precipitating renal tubular epithelial cell pyroptosis.^13^, ^14^, ^15^ GSDMD presents as a potential therapeutic target for AKI treatment. However, current clinical drugs that can efficaciously inhibit GSDMD‐dependent pyroptosis are scant and warrant further exploration.^16^

In our quest for potential AKI therapeutics via the GSDMD pathway, we examined multiple drug libraries using our in vitro cell assays. This led to the identification of a series of natural small molecule drugs that could potentially inhibit the GSDMD signaling pathway. Notably, stevioside stood out, showcasing a superior reno‐protective effect in vitro. Extracted from stevia leaves, stevioside, besides being a natural sweetener, also offers a plethora of pharmacological benefits including anti‐hypertension, antioxidant, anti‐inflammatory effects, and immune modulation.^17^ Recent studies revealed that stevioside can downregulate the expression and production of various pro‐inflammatory cytokines, including interleukin 6 (IL‐6), tumor necrosis factor *α* (TNF‐α) and IL‐1β, and upregulate anti‐inflammatory cytokines like IL‐10 and transforming growth factor β1 (TGF‐β1) in lipopolysaccharide‐induced macrophages, thereby reducing inflammatory responses in vitro and in vivo through multiple signaling pathways.^18^, ^19^ Additionally, acute and subacute toxicity studies have demonstrated the low toxicity of stevioside in various experimental mice models.^20^, ^21^ Integrating our findings with existing research, we postulate that stevioside may attenuate the post‐AKI inflammatory response, suggesting its potential as a nephroprotective agent.

Consequently, this study endeavors to elucidate the nephroprotective prowess of stevioside in AKI treatment. We also aim to unravel the GSDMD signaling pathway as a plausible mechanism underpinning the therapeutic effects induced by stevioside, leveraging cisplatin and IR induced cell and animal models.

## 2 EXPERIMENTAL METHODS

### 2.1 Materials

Stevioside (Ste, Purity: 99.52%, CAS NO.: 57817‐89‐7), Madecassoside (MA, Purity: 99.75%, CAS NO.: 34540‐22‐2) and Arachidonic acid (AA, Purity: 99.78%, CAS NO.: 506‐32‐1) all purchased from Topscience (Shanghai, China). The fetal bovine serum, Dulbecco's Modified Eagle Medium/Nutrient Mixture F‐12 (DMEM/F12) medium and Roswell Park Memorial Institute (RPMI) 1640 medium were purchased from Gibco (Grand Island, USA). ITS was from Invitrogen (Carlsbad, CA, USA), and mouse Epidermal Growth Factor (mEGF) was provided by R&D Systems (Emeryville, CA, USA). Nicotinamide adenine dinucleotide phosphate (NAPDH), 2′, 7′‐Dichlorofluorescein diacetate (DCFH‐DA), Lactate dehydrogenase (LDH) cytotoxicity assay kit, radioimmunoprecipitation assay (RIPA) Lysis Buffer and BCA Protein Assay Kit were from Beyotime (Shanghai, China). Phosphatase and protease inhibitor were provided from Boster (Wuhan, China). Annexin V‐Alexa Fluor 647/PI Apoptosis Detection Kit was from Yeasen (Shanghai, China). Hematoxylin and eosin were from Servicebio (Wuhan, China). Diaminobenzidine (DAB) was from ZSGB‐BIO (Beijing, China). The antibodies used for Immunohistochemistry (IHC) were as follows: fibronectin (cat. no. ab2413, Abcam, Cambridge, MA, USA) and collagen I (cat. no. ab34710, Abcam, Cambridge, MA, USA). The antibodies used for western blotting were as follows: GSDMD (Abcam, Cambridge, MA, USA), GSDMD‐NT (Abcam, Cambridge, MA, USA), GAPDH (Proteintech, Wuhan, China) and *β*‐actin (Proteintech, Wuhan, China). Mouse IL‐1β ELISA Kit (cat. no. FMS‐ELM002, Nanjing, China) and Mouse IL‐6 ELISA kit (cat. no. FMS‐ELM006, Nanjing, China) were provided by the FCMACS (Nanjing, China). Human Proximal tubular epithelial cells (HK‐2) was provided by the Cell Bank of Shanghai Institutes for Biological Sciences (Shanghai, China).

### 2.2 Cell culture and treatment

HK‐2 were obtained from the Cell Bank of the Chinese Academy of Science (Catalog no. SCSP‐511, Shanghai) and cultured in DMEM. Immortalized BM‐derived macrophages (iBMDM) cells were kindly provided by Professor Cun‐jin Zhang's laboratory at Nanjing University and cultured in RPMI 1640 medium. All mediums mentioned above were supplemented with 10% fetal bovine serum, 1% penicillin and streptomycin in a humidified incubator at 37°C with 5% CO_2_ level. The HK‐2 cells and iBMDM cells were both cultivated at least 3 generations after resuscitation for subsequent experiments.

### 2.3 Extraction and culture of primary renal tubular epithelial cells

Kidneys from CD1 pups, about 1 week old, were harvested in a sterile environment and placed in pre‐cooled saline. The kidney tissue was ground and passed through 50 and 150 mesh sieves successively to collect the tissue, and the supernatant was discarded after centrifugation at 1000 g for 5 min. Collagenase I diluted with basal medium (DMEM/F12) was added to the pellet, which was placed in a 37°C incubator for 25 min, and cells were mixed by shaking every 5 min. The supernatant was removed by centrifugation at 1000 g for 5 min, then D‐F12 complete medium containing 10% fetal bovine serum, 1% penicillin and streptomycin, 1% ITS, and 10 ng/mL mEGF was added. PTCs were pipetted evenly and inoculated in cell culture dishes. The complete medium was replaced 24 h later. Cells at density 80%–90% were treated with H_2_O_2_ for 24 h, added with Stevioside (25 μM), MA (10 μM) or AA (10 μM) for the indicated time, and subjected to the subsequent experiments.

### 2.4 High‐throughput drug screening

iBMDM cells (10^4^ cells per well) were seeded on 96‐well plates and cultured in an incubator (95% air, 5% CO_2_) at 37°C for 24 h. The iBMDM cells were treated with LPS for 3 h, then two thousand three hundred and sixty‐four small molecules were added into each well at a final concentration of 10 μM for 6 h. Subsequently, the iBMDM cells were treated with Nigericin for 1 h. According to the manufacturer's instructions, the CellTiter Glo ® Luminescent Cell Viability Assay kit was used for detecting the viability of iBMDM cells. Select cost‐effective drugs based on cell viability.

### 2.5 LDH release assay

LDH is indicative of the loss of cell membrane integrity and thus represents cell death. In accordance with the manufacturer's instructions, the LDH release agent was first added to the control sample with the maximum enzyme activity prepared in advance and allowed to be lysed wholly. Then, a 96‐well plate was added with the prepared LDH working solution and incubated at 37°C for 30 min. Lastly, the plate was measured spectrophotometrically at 600 nm with a microplate reader (Synergy H1 Hybrid Reader). The values shown are the percentages of total LDH (intracellular plus supernatant LDH).

### 2.6 Cell viability assay

We used the Annexin V‐Alexa Fluor 647/PI Apoptosis Detection Kit to examine cell viability. We divided cells into three groups with different treatments and digested cells from the 6‐well plates with trypsin containing Ethylene Diamine Tetraacetic Acid (EDTA), transferred them to 1.5‐mL Eppendorf tubes, and centrifuged them at 4°C and 300 g for 5 min to collect the cells. We washed cells twice with pre‐chilled PBS, then added 250 μL 1×binding buffer to resuspend the cells and adjust the concentration to 1 × 10^6^/mL. Next, we took 100 μL cell suspension, added 5 μL Annexin V‐Alexa Fluor 647 and 10 μL PI, and mixed them gently to react at room temperature for 15 min without light. Finally, we added 400 μL PBS and analyzed the sample by flow cytometry.

CellTiterGlo® Luminescent Cell Viability Assay was a homogeneous assay for detecting the number of viable cells in culture by quantitatively measuring ATP, which was an indicator of viable cell metabolism. The reagent (CellTiter‐Glo® reagent) was directly added to the cultured cell 96‐well plate containing serum, shaken and mixed for 5 min, and incubated at room temperature for 30 min. The GloMax luminescence detector was used to detect the reading.

Added methylthiazolyldiphenyl‐tetrazolium bromide (MTT) reagent to the treated 96‐well plate for 2 h, and an equal volume of dimethyl sulfoxide (DMSO) was added and incubated at 37°C for 10 min. The absorbance was detected at a wavelength of 490 nm by a Microplate reader.

### 2.7 Reactive oxygen species

After washing the cells with DMEM/F12 medium, the reactive oxygen species probe DCFH‐DA diluted with the medium was added and then placed in an incubator with CO_2_ at 37°C for 20 min. After repeated washing, the cells were observed and photographed under a fluorescence microscope.

### 2.8 Animals

Mice were housed under specific pathogen‐free conditions at the Laboratory Animal Center of Nanjing University and randomly assigned to experimental animal groups. C57BL6 mice (SPF grade, male, 6–7 weeks old, 20–25 g) were purchased from Nanjing Qinglongshan animal farm. The present study was approved by the Institutional Animal Care and Use Committee, Drum Tower Hospital, Medical School of Nanjing University, in accordance with the institutional guidelines for the care and use of laboratory animals. Animal experiments were performed at Nanjing University and Nanjing Drum Tower Hospital.

### 2.9 Mouse model

Cisplatin‐induced AKI mouse model: Male C57BL6 mice (20–25 g, aged 6–7 weeks) were intraperitoneally injected with a single dose of cisplatin at 40 mg/kg, and the treated group's mice were infused with 50 mg/kg stevioside every 24 h and the renal cortex was collected 48 h later.

IR‐induced AKI mouse model: Mice were anesthetized with 5% pentobarbital for pain relief. Mice were placed in a water bath for the whole experiment, and the core body temperature was maintained at 37°C. Mice were anatomized from the abdominal cavity. The unilateral kidney was exposed and the left renal pedicle was clamped with an arterial clamp to induce renal ischemia. After 50 min of clamping, the arterial clips were released for reperfusion. Renal blood flow was observed to return to the original color, indicating successful reperfusion, and the renal cortex was collected after 24 h. Before cisplatin and IR induction, mice in the experimental group were infused with 50 mg/kg stevioside every 24 h for 7 consecutive days, and the other mice were injected with phosphate buffered saline 100 μL per day as a control; kidneys in the control group were removed without any treatment.

### 2.10 Western blot analysis

After euthanizing the mice, approximately 0.1 g of kidney tissue was taken and placed in a grinding tube. 400–500 μL of RIPA lysis solution containing protease inhibitors and phosphatase inhibitors was added to each tube, and the kidney tissue was lysed by the SpeedMill PLUS homogenizer (Analytick Jena, Germany) kidney tissue. The cellular and tissue proteins were extracted in RIPA lysis buffer for 15 min on ice. The lysates were collected after centrifugation at 12,000  rpm for 15 min at 4°C.

The BCA protein detection kit was used to detect the protein level. The protein was mixed with 5× loading buffer and denatured by boiling at 99°C for 5 min. The mixture was separated by sodium dodecyl sulfate polyacrylamide gel electrophoresis (SDS‐PAGE) and transferred to a polyvinylidene fluoride membrane. The membrane was blocked in 5% skim milk for 1 h and incubated with anti‐GSDMD and anti‐GSDMD‐NT at 4°C overnight. After washing with PBS‐T, the membrane was incubated with the appropriate secondary antibody for 1 h at room temperature. Immunoreactive bands were visualized and analyzed using an enhanced chemiluminescence luminescent solution. Specific protein levels were normalized to that of *β*‐actin or GAPDH.

### 2.11 Immunohistochemistry and histology

The renal sections were deparaffinized and rehydrated, incubated with 3% hydrogen peroxide for 15 min, and then heated with sodium citrate buffer for antigen retrieval. After blocking with 5% goat serum albumin, the sections were incubated with anti‐fibronectin and anti‐collagen overnight at 4°C, followed by the secondary antibody. The sections were stained with DAB chromogenic agent and re‐stained with hematoxylin. In addition, sections were stained with hematoxylin and eosin to evaluate the degree of inflammatory cell infiltration. Sections were viewed under a fluorescence microscope and the immunoreactivity was quantified using Image‐Pro Plus v.6.0 (Media Cybernetics).

### 2.12 RNA extraction and quantitative RT‐PCR

Total RNA was extracted from kidney tissue or cells using Trizol reagent. Total RNA was quantified and underwent reverse transcription into cDNA with PrimeScript RT Master Mix kit for qPCR. Real‐time quantitative PCR for *β*‐actin, *IL‐10*, *IL‐18* and *TNF‐α* involved the following primers to amplify genes:

mouse *β*‐actin forward, 5′‐GGCTGTATTCCCCTCCATCG‐3′
reverse, 5′‐CCAGTTGGTAACAATGCCATGT‐3’; mouse *IL‐10* forward 5′‐GCTCTTACTGACTGGCATGAG‐3’
reverse, 5′‐CGCAGCTCTAGGAGCATGTG‐3’; mouse *IL‐18* forward 5′‐ACCTCCAGCATCAGGACAAAG‐3’
reverse, 5′‐TGTACAGTGAAGTCGGCCAAAG‐3’; mouse *TNF‐α* forward 5′‐GCGACGTGGAACTGGCAGAAG‐3’
reverse, 5′‐GCCACAAGCAGGAATGAGAAGAGG‐3’.

The raw data (Ct values) were analyzed using the comparative Ct method.

### 2.13 Enzyme linked immunosorbent assay (ELISA)

IL‐1β levels in cell culture supernatant and IL‐6 levels in kidney tissue were detected using the Mouse IL‐1β ELISA Kit and Mouse IL‐6 ELISA kit. The procedures followed the manufacturer's protocols. Samples and standards of different concentrations were added to the corresponding wells, then the reaction wells were sealed with sealing tape, incubated at 37°C for 90 min, and washed four times. Biotinylated antibody working solution was added and the solution was incubated at 37°C for 60 min, and the plate was washed four times. The enzyme conjugate working solution was added and the solution was incubated at 37°C for 30 min. After washing the plate four times, the color reagent was added and the solution was incubated at 37°C for 15 min. Finally, the stop solution was added, and the OD450 value was measured right after mixing it immediately.

### 2.14 Statistical analysis

We used GraphPad prism8 for all statistical analysis. All data are presented as mean ± standard error of the mean (SEM) or standard deviation (SD). Student's *t* test was used for comparisons between experimental groups. One‐way ANOVA and Newman‐Keuls Multiple Comparison Test were used to test the comparison between multiple groups. *p* < 0.05 was considered statistically significant.

## 3 RESULTS

### 3.1 Stevioside identified as a potent nephroprotective molecule

**FIGURE 1:**
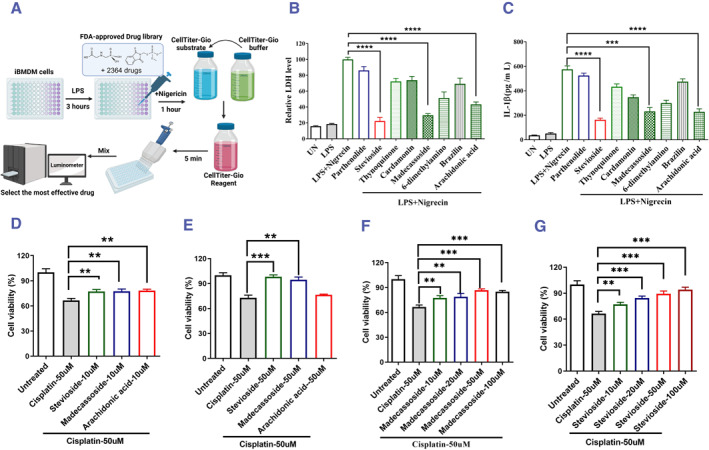
Therapeutic impact of stevioside on renal cells. (A) The schematic diagram of the drug screening protocol. (B–C) Screening of small molecule inhibitors targeting GSDMD signaling assessed by LDH release and IL‐1β levels. (D) ATP‐based cell viability of HK‐2 cells post 50 μM cisplatin treatment with concurrent 10 μM inhibitor treatment for 24 h. (E) ATP‐based cell viability of HK‐2 cells posts 50 μM cisplatin treatment with concurrent 50 μM inhibitor treatment for 24 h. (F–G) Concentration gradient study of stevioside and madecassoside post 50 μM cisplatin treatment. The data all represent measurement data presented as the mean ± SEM, *n* = 3. *p < 0.05, **p < 0.01, ***p < 0.001.

A drug screening assay was performed by incubating over a thousand small molecules from the Drug Bank incubated with LPS and Nigericin‐stimulated iBMDM (Figure 1A). A subset of natural molecules emerged that could effectively inhibit LDH release and IL‐1β levels. Using these markers to monitor GSDMD signaling inhibition, stevioside, madecassoside, and arachidonic acid stood out significantly (Figure 1B,C).

To assess their protective attributes in the renal cell damage induced by cisplatin, these molecules were tested against renal cell damage caused by cisplatin. The combined effect of 50 μM cisplatin and 10 μM of these three drugs for 24 h, the results indicated that these three drugs could reveal notable protection against HK‐2 cell death (Figure 1D). Further investigations showed that only stevioside and madecassoside remained effective at high concentrations of 50 μM (Figure 1E). Stevioside's protective nature was also identified to be dose‐dependent (Figure 1F,G), making it a prime candidate for deeper exploration.

### 3.2 Stevioside attenuates H_2_O_2_‐induced renal tubular epithelial cell injury

**FIGURE 2:**
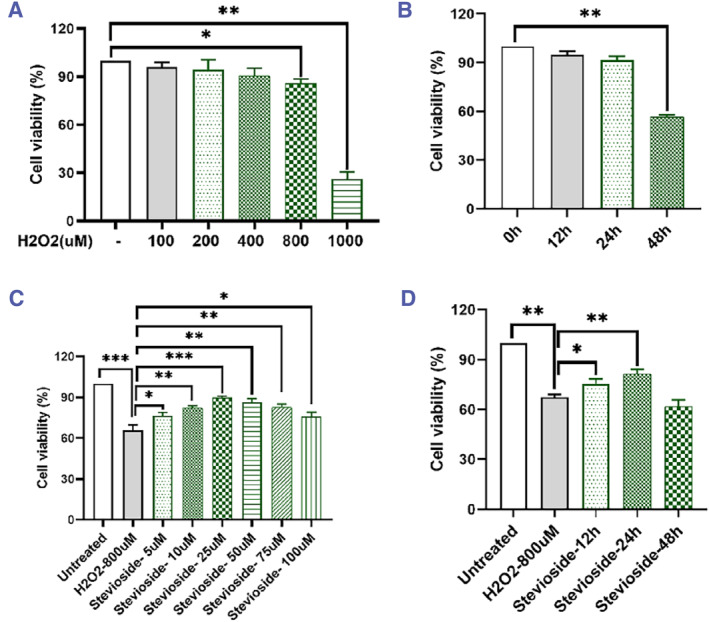
Stevioside's protection against H_2_O_2_‐induced PTC Injury. (A) Viability of PTCs following varying H_2_O_2_ concentrations using the MTT assay. (B) MTT‐based viability assessment of PTCs post H_2_O_2_ treatment across different time intervals. (C) MTT evaluation of stevioside's protective role against H_2_O_2_‐induced PTCs injury at diverse concentrations. (D) MTT assessment of 25 μM stevioside's protective duration against H_2_O_2_‐induced PTCs damage. The data all represent measurement data presented as the mean ± SEM, *n* = 3. *p < 0.05, **p < 0.01, ***p < 0.001.

An MTT assay determined the optimal H_2_O_2_ concentration and duration causing significant PTC damage. The results showed that at 0–800 μM, the cell survival rate showed a slow decreasing trend with increasing concentration, while at 800–1000 μM, the cell survival rate suddenly decreased to less than 50% (Figure 2A). Then, 800 μM H_2_O_2_ was screened for an action time, and the results showed that from 0 to 24 h, the cell survival rate showed a slow decreasing trend with the increase of action time. However, from 24 to 48 h, the cell survival rate suddenly decreased to 55%–60%, causing significant damage to the cells (Figure 2B). Therefore, the optimal damage time for PTCs was selected as 24 h of H_2_O_2_ action.

Notably, On the basis of treating with H_2_O_2_ (800 μM) for 24 h, we further examined the optimal concentration of stevioside to treat PTC damage induced by H_2_O_2_. The results showed that at 0–25 μM, the cell survival rate increased with the increase of concentration, while it decreased when it exceeded 25 μM. In addition, when treated with stevioside (25 μM) for 24 h, it can significantly rescue H_2_O_2_ induced PTC damage, reaching the highest point of cell survival rate (Figure 2C,D).

### 3.3 Stevioside mitigates GSDMD‐dependent inflammatory response

**FIGURE 3:**
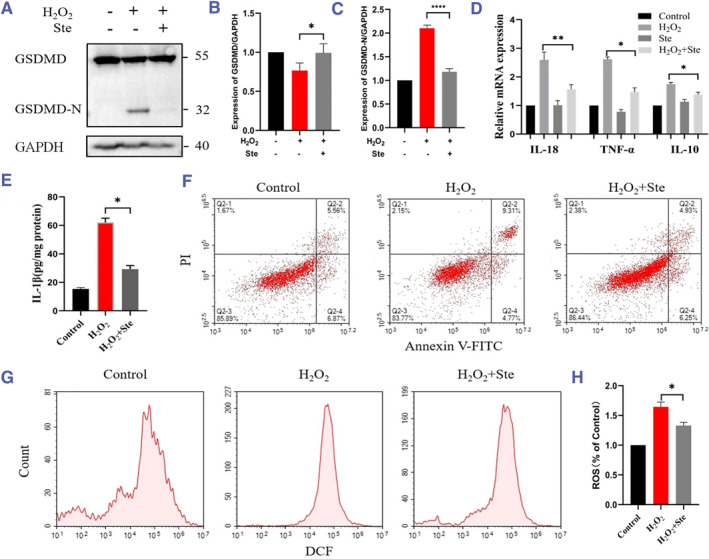
Stevioside's modulation of GSDMD pathway and associated inflammatory responses in PTCs. (A–C) Western blot examination of GSDMD and GSDMD‐NT expressions in PTCs post H_2_O_2_ and stevioside treatments. (D) RT‐qPCR analysis of IL‐10, IL‐18, and TNF‐α mRNA levels. (E) ELISA evaluation of IL‐1β expression post H_2_O_2_ and stevioside treatments. (F) Flow cytometry insights into GSDMD‐mediated pyroptosis changes post stevioside intervention. (G‐H) ROS level alterations post treatments. Data are mean ± SEM (*n* = 3). *p < 0.05, **p < 0.01, ****p < 0.0001. IL, interleukin; TNF, tumor necrosis factor; Ste, Stevioside.

To establish stevioside's efficacy in counteracting renal injury via the GSDMD pathway, a mouse PTCs injury model was exposed to H_2_O_2_. The protein expression of GSDMD was correspondingly downregulated after H_2_O_2_ treatment, but the cleavage of GSDMD‐NT was increased and the expression level was about 200% that of the control group. In addition, the production of GSDMD‐NT was significantly inhibited after stevioside treatment and reduced to a level close to the control group. These results suggested that stevioside could inhibit the activation of GSDMD (Figure 3A–C). Additionally, inflammatory factors like *IL‐10*, *IL‐18*, and *TNF‐α* were elevated post‐injury but showed significant reductions with stevioside treatment (Figure 3D). Stevioside also notably curtailed GSDMD‐associated IL‐1β expression, that is, the IL‐1β level decreased to 50% after treatment with stevioside compared to the injury group (Figure 3E). Besides, GSDMD‐mediated apoptosis was also reduced after stevioside treatment (Figure 3F), and the ROS levels in injured cells were significantly decreased (Figure 3G,H). Our findings suggested that stevioside could significantly inhibit H_2_O_2_‐induced injury of renal tubular epithelial cells and the release of GSDMD‐dependent inflammatory factors.

### 3.4 Stevioside exerts significantly protective effects in 2 mouse models of AKI

**FIGURE 4:**
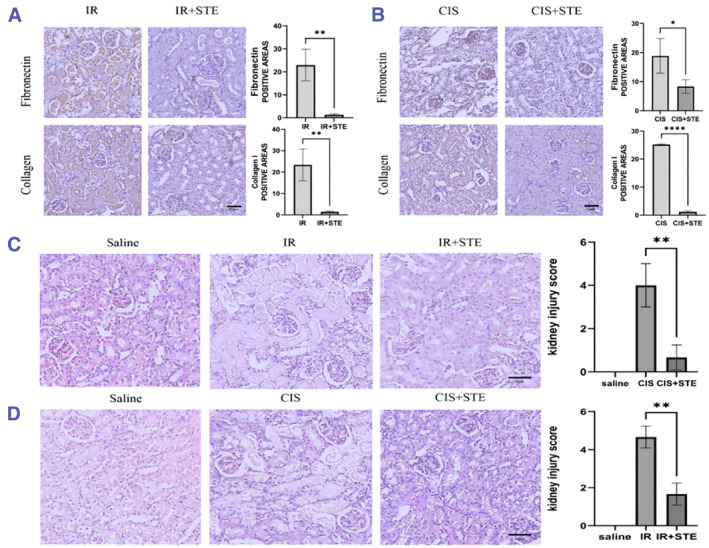
Stevioside's protective action in mouse AKI Models. (A–B) Expression levels of fibronectin and collagen I post IR and stevioside interventions. (C–D) Comparative assessment of cast formation, tubular necrosis, brush border loss, and tubular dilation across treatment groups. Data are mean ± SEM (*n* = 3). *p < 0.05, **p < 0.01, ****p < 0.0001.

To ascertain the therapeutic benefits of stevioside in vivo, 2 mouse AKI models were examined. Post kidney extraction, the expression of fibronectin and collagen I was both high in IR‐induced and cis‐induced kidney injury models, while reduced expressions of fibronectin and collagen I were observed in the presence of stevioside (Figure 4A,B). Concurrently, the degree of cast formation, tubular necrosis, loss of brush border and tubular dilation were notably reduced after stevioside treatment (Figure 4C,D). These results suggest that stevioside can significantly delay the progression of AKI in mice.

### 3.5 Stevioside counteracts renal injury by targeting GSDMD signaling

**FIGURE 5:**
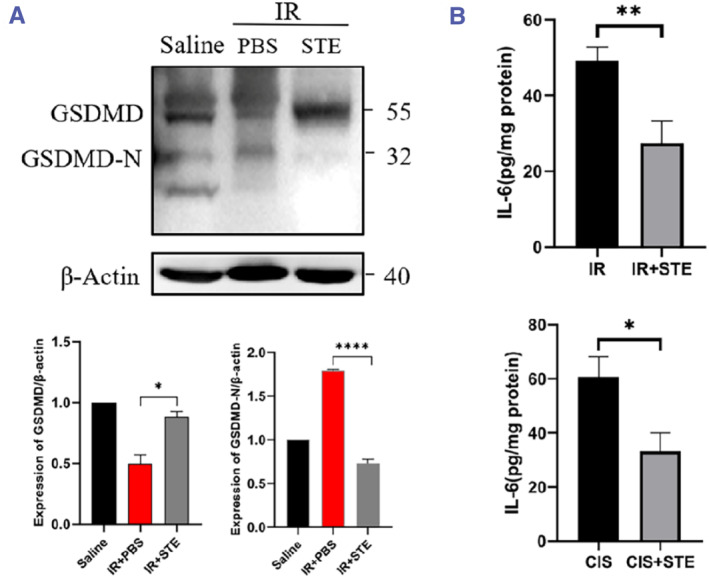
Stevioside's regulatory role on GSDMD signaling in mouse renal tissues. (A) Western blot insights into GSDMD and GSDMD‐NT expressions in mouse kidney tissues post injury and stevioside treatment. (B) ELISA analysis of IL‐6 concentration post treatment. Data are mean ± SD (*n* = 3). *p < 0.05, **p < 0.01, ****p < 0.0001.

Our in vivo studies corroborated stevioside's role in delaying AKI and offering renal protection. To investigate whether stevioside acts through GSDMD in vivo, tissue proteins were extracted from primary mouse kidneys for follow‐up experiments. While the protein expression of GSDMD was decreased, the production of GSDMD‐NT increased nearly 1.7 times after renal injury (Figure 5A). Furthermore, the expression of GSDMD‐NT was significantly decreased to half of the AKI group in mice with renal injury along with stevioside intragastric administration for 1 week. In addition, the expression of the inflammatory factor IL‐6 was decreased to 50%–60% after stevioside treatment (Figure 5B). The above results show that stevioside can delay kidney injury in mice by inhibiting the GSDMD signaling pathway; it can inhibit the activation of GSDMD and reduce the release of inflammatory factors both in vitro and in vivo.

## 4 DISCUSSION

Acute Kidney Injury (AKI) is a form of rapid‐onset renal failure precipitated by ischemic or nephrotoxic damage, leading to various negative prognostic outcomes.^2^, ^7^, ^8^ Key features of AKI include cellular death and heightened inflammation.^22^ Recent research has spotlighted the pronounced upregulation of GSDMD in AKI and its role in mediating pyroptosis and releasing inflammatory cytokines, underscoring its potential as a drug target.^10^, ^23^, ^24^ Leveraging artificial intelligence, we conducted a comprehensive screening of various drug libraries, unearthing a selection of natural small molecules capable of inhibiting the GSDMD signaling pathway. Notably, our preliminary low‐dose screening revealed steviosides potential in counteracting cisplatin‐induced death in HK‐2 cells in a concentration‐dependent manner. Subsequent experiments validated the effectiveness of stevioside in reducing GSDMD expression post‐injury, curtailing the cleaved GSDMD‐NT and inflammatory markers. The reduction of apoptosis and ROS linked to GSDMD further substantiates this finding. Validation in both cisplatin and ischemia‐reperfusion AKI mouse models further demonstrated stevioside's renal protective properties. Our results indicate stevioside's efficacy in attenuating casting formation, renal tubular necrosis, and other markers of renal injury. Moreover, our in vivo studies on kidney histone proteins reveal a significant drop in GSDMD‐NT and IL‐6 expressions post‐stevioside administration, making this study a pioneering effort in establishing stevioside as a therapeutic agent for AKI via GSDMD pathway inhibition.

As a widely recognized food sweetener, stevioside has documented pharmacological benefits spanning antidiabetic, antihypertensive, and renoprotective actions.^18^, ^25^, ^26^ In cisplatin‐induced mouse AKI, stevioside inhibits oxidative stress, inflammation, and apoptosis by regulating the expression of cisplatin‐activated extracellular signal‐regulated kinases 1 and 2 and signal transducer and activator of transcription 3 (STAT3), thereby reducing the nephrotoxicity of cisplatin.^27^ In addition, studies of renal fibrosis have found that stevioside can inhibit unilateral ureteral obstruction‐induced renal fibrosis in mice, and its anti‐fibrotic mechanism is related to the activation of peroxisome proliferator‐activated receptor gamma, which can increase the expression of STAT3 and TGF‐β1 mediated by nuclear factor kappa‐B and the downregulation of Smad blocks the Smad‐mediated fibrosis signaling pathway.^28^ Currently, stevioside has been partially studied in the kidney,^29^, ^30^ but its capacity to modulate AKI via GSDMD regulation remains an uncharted territory. With H_2_O_2_‐induced renal tubular epithelial cell injury, we found that stevioside could inhibit GSDMD activation, thus leading to the release of inflammatory factors and apoptosis. In 2 mouse models of AKI induced by cisplatin and ischemia‐reperfusion, we also verified that stevioside could reduce the release of inflammatory factors and has a good renal protective effect.

Gasdermins are a family of pore‐forming effector proteins that contain a cytotoxic N‐terminal domain and a C‐terminal repressor domain linked by a flexible linker.^31^ When cells become infected or cancerous, gasdermin punctures the cell membrane to form pores, and a large number of inflammatory cytokines leak out of the pores and trigger pyroptosis.^30^, ^32^ Gasdermin‐induced pyroptosis plays an important role in many genetic and (auto) inflammatory diseases as well as in cancer.^33^, ^34^, ^35^ In addition to GSDMD, the gasdermin family contains the members GSDMA, GSDMB, GSDMC, GSDME (also known as DFNA5), and PJVK (also known as DFNB59). Except for DFNB59, other components have been found related to pyroptosis.^30^ Caspases were found to be cleaved at the central junction of GSDMD to generate a 31‐kDa N‐terminus and a 22‐kDa C‐terminus. GSDMD's N‐terminus alone can induce apoptosis.^33^ Recent research has found that GSDMD is significantly upregulated in AKI; therefore, GSDMD is a highly attractive candidate target for drug development.^10^, ^23^ The research group previously obtained through high‐throughput screening that stevioside can inhibit the GSDMD signaling pathway. Therefore, whether steviosides have the potential to become small molecule drugs with renal protective effects based on the GSDMD signaling pathway is a further exploration direction of this study.

Altogether, our data demonstrate that stevioside can reduce the release of inflammatory factors by inhibiting the activation of the GSDMD signaling pathway, prevent cell pyroptosis, and provide good renal protection. Our study offers pivotal insights into the role of a specific inhibitory molecule in AKI progression, thereby pointing towards a novel therapeutic approach. However, a notable limitation remains: our study did not venture into the synthesis of stevioside analogs, which could potentially enhance renal protection. Furthermore, the validation with clinical samples is pending, which might be an avenue for future investigations. In sum, our findings affirm stevioside's capability to mitigate inflammation via GSDMD pathway inhibition, averting cell pyroptosis and offering significant renal protection. This presents a promising avenue for novel clinical interventions for AKI.

## AUTHOR CONTRIBUTIONS

Ruochen Qiao and Hui Wang conceived the idea and designed the experiment; Ruochen Qiao, Dasheng Li, Yu Yang, Jiaxin Shu, and Xiang Song conducted experiments; Yu Yang and Jiaxin Shu performed data analysis; Ruochen Qiao and Li Lu drafted and revised the manuscript; Xiaozhi Zhao and Li Lu revised the final version of the manuscript; Xiaozhi Zhao provides fund support. All authors take responsibility for the integrity of the data analysis.

## CONFLICT OF INTEREST STATEMENT

The authors declare that there are no competing interests.

## ETHICS STATEMENT

The authors are accountable for all aspects of the work in ensuring that questions related to the accuracy or integrity of any part of the work are appropriately investigated and resolved.

## Data Availability

The data that support the findings of this study are available from the corresponding author upon reasonable request.
